# Reply to Noori et al., “A Complex Scenario of Nonsteroidal Anti-inflammatory Drugs Induced Prostaglandin E2 Production and Gut Microbiota Alteration in Clostridium difficile-Infected Mice”

**DOI:** 10.1128/mBio.03142-19

**Published:** 2020-01-28

**Authors:** Damian Maseda, Joseph P. Zackular, David M. Aronoff

**Affiliations:** a Department of Microbiology, Perelman School of Medicine, University of Pennsylvania, Philadelphia, Pennsylvania, USA; b Department of Pathology and Laboratory Medicine, Perelman School of Medicine, University of Pennsylvania, Philadelphia, Pennsylvania, USA; c Division of Protective immunity, The Children’s Hospital of Philadelphia, Philadelphia, Pennsylvania, USA; d Department of Pathology, Microbiology, and Immunology, Vanderbilt University School of Medicine, Nashville, Tennessee, USA; e Department of Medicine, Vanderbilt University School of Medicine, Nashville, Tennessee, USA

**Keywords:** *Clostridioides difficile*, cyclooxygenase, microbiome, prostaglandins

## REPLY

We thank Noori and colleagues for their interest in our recent work and appreciate their interpretation of the data from our study in the context of other existing data (1). They underscore an interesting finding in our study: colon levels of the lipid mediator prostaglandin E2 (PGE_2_) paradoxically increased during Clostridioides difficile infection (CDI) following a brief exposure to the nonsteroidal anti-inflammatory drug (NSAID) indomethacin, which is known to inhibit PG synthesis (2). This increase in tissue PGE_2_ was associated with marked tissue inflammation, upregulation of the inducible PGE synthase enzyme encoded by the *PTGES* gene, and suppression of the PGE_2_-inactivating enzyme 15-PG dehydrogenase (15-PGDH), encoded by the *HPGD* gene (2).

Noori and colleagues note that in some experimental settings the pharmacological inhibition of PGE_2_ synthesis has resulted in induction of *HPGD* expression. It should be noted that such studies have described this phenomenon in cancer cell lines, while in contrast our study used freshly isolated murine cecal and colon tissues. The authors of this letter also suggest that in the setting of CDI the NSAID indomethacin might itself induce *HPGD* expression, rather than suppress it, which they speculate would result in elevated PGE_2_ production (1). This is a confusing position, since increased 15-PGDH would be expected to reduce PGE_2_ levels through oxidative metabolism (3), as implied in Fig. 1 of the authors’ letter. We cannot fully explain the suppression of *HPGD* expression in our model but would like to emphasize that this occurred several days after the brief NSAID exposure, at a time when colonic inflammation was high, as were tissue PGE_2_ levels. There is also the possibility of a discordance between the transcript and protein levels of 15-PGDH, and we did not measure the latter. Regardless, the increased level of PGE_2_ found in the colon during infection (our Fig. 3H) is consistent with the findings of others, reviewed by Noori and colleagues in their letter, that tissue levels of PGE_2_ are increased during CDI.

The authors note that, in contrast to our *mBio* study, in another mouse model of CDI indomethacin use was associated with reduced colon PGE_2_ levels (4). That study, which involved members of our research group, differed fundamentally from our *mBio* study in that C. difficile-infected mice were treated with indomethacin throughout their infection and tissue PGE_2_ levels were assessed during NSAID therapy (4). In our *mBio* study, we assessed tissue PGE_2_ levels several days after withdrawal of the NSAID (2).

Noori and colleagues conclude their letter with the idea that indomethacin might have induced gut microbiota dysbiosis in association with CDI-induced proinflammatory changes that triggered the induction of inducible cyclooxygenase 2 (COX-2) enzyme and thereby enhanced the production of PGE_2_. This idea is consistent with our findings, with the exception that we did not observe induction of COX-2 but rather observed an increase in *PTGES* mRNA, which would have achieved a similar result (elevated PGE_2_).

Eicosanoids such as PGE_2_ are clearly important in gastrointestinal homeostasis, and understanding the complex mechanisms underlying the therapeutic and detrimental effects of NSAIDs in this context is important, given the significant popularity of this class of drugs in clinical medicine.
